# Prevalence of and risk factors for different measures of low back pain among female nursing aides in Taiwanese nursing homes

**DOI:** 10.1186/1471-2474-8-52

**Published:** 2007-06-25

**Authors:** Chao-Kang Feng, Mei-Lien Chen, I-Fang Mao

**Affiliations:** 1 Institute of Public Health, National Yang-Ming University No. 155, Li-Nong Street, Sec. 2., Taipei, Taiwan; 2 Department of Healthcare Administration, Hung-Kuang University No. 34, Chung-Chie Rd., Shalu, Taichung County, Taiwan; 3 Institute of Environmental Health Sciences, National Yang-Ming University No. 155, Li-Nong Street, Sec. 2., Taipei, Taiwan

## Abstract

**Background:**

Although low back pain (LBP) among nursing staff, especially in nursing aides (NAs), has been a major health problem around the world, there is limited information on its prevalence in Taiwan. In addition, various measurements have been used to determine LBP; understanding the risk factors for each measurement of LBP is essential for prevention. This study aimed to assess the prevalence of and risk factors for different measures of LBP among NAs in Taiwan.

**Methods:**

A cross-sectional study was conducted among 244 female NAs from 31 nursing homes in central Taiwan. A self-administered questionnaire, including the Nordic questionnaire and the Karasek's job content questionnaire, was used to collect data regarding five different measures of LBP and about demographic, physical and psychosocial factors. Also, on-site observation at the workplace was conducted to measure the frequency of five high risk patient-handling tasks.

**Results:**

Based on the subjects' reports on the previous twelve months, the prevalence rates for pain lasting for at least one day, seeking of medical care, intense pain, sick leave, and chronic pain were 66.0%, 43.9%, 38.1%, 10.7%, and 8.6%, respectively. While multiple logistic regression analyses indicated that the risk factors varied with different measures of LBP, at least one high risk patient-handling task and one psychosocial factor were observed to be associated each LBP related measure. Three risk factors, including manual transfer of patients between bed/wheelchair and bath cart, perceived physical exertion, and psychological demands, were consistently associated with different measures of LBP. Besides, age was found to be associated with an increased risk of only chronic pain.

**Conclusion:**

The prevalence of LBP among NAs in Taiwan is high and should be actively addressed. Certain manual patient-transfer tasks and psychological demands seemed to play more important roles in severe LBP (such as care seeking, intense pain, and sick leave) than in minor LBP (pain lasting for at least one day). Because different LBP related measures might be involved with different etiological risk factors, any LBP reduction interventions that aim to improve ergonomic and psychosocial work environments for NAs should take this information into consideration.

## Background

Because many nursing home residents depend heavily on nursing aides (NAs) in most of their daily activities which are usually physically demanding, numerous studies have reported that NAs working at nursing homes are likely to suffer more frequently from low back pain (LBP) than registered nurses [1-3]. Evidence has shown that more patient dependence would lead to a higher prevalence of LBP for his/her caregivers [4]. Previous empirical studies have also suggested that NAs who usually perform physically demanding tasks in nursing homes were at an increased risk of LBP [5,6].

Many previous studies have been conducted to identify the roles of individual and occupational factors as possible causes of LBP among nursing staff. Some previous studies have shown that ergonomic risk factors, including physical exertion at work, frequent bending and twisting, heavy lifting and other patient-handling tasks, all play important roles in contributing to the occurrence of LBP [7-9]. Some other studies have indicated an association between work-related psychological and psychosocial factors and risks of LBP among nursing staff [9-11], but such observations were not reproduced by other studies [12,13]. On the other hand, study results regarding the association between demographic characteristics and risk of LBP among nursing staff have been very inconsistent [3,7,10,14]. Such inconsistency could be due to inadequate statistical power in certain studies, or due to the fact that LBP is a vague term which lacks consensual definition and conceals many different symptomatic conditions [15,16]. It has been noted that various measures were used to determine and to characterize LBP. These measures include duration of LBP [8,9,12], pain severity [6,10,11] and certain indicators of the consequences of LBP such as the seeking of health care [17] and sick leave for LBP [6,12,18].

Although LBP is a major health problem among nursing staff, most previous studies have explored only one [7-10] or two [2,6,19] measures of LBP and their associated risk factors. The severity of LBP has rarely been taken into consideration in these studies. Hence, it may be difficult to distinguish the risk factors for minor LBP from those for severe LBP. Ozguler and associates suggested that future studies should pay more attention to different measures of LBP [16]. According to our knowledge, only two studies examined over three measures of LBP simultaneously [16,20]. But none of those studies were conducted among nursing populations.

Assessment of physical activities of nursing staff using the video taping technique can be extremely difficult, mainly because the physical activities of nursing work are not cyclic and are not limited to a specific work area. Furthermore, there is concern regarding patient's privacy. Thus, most previous studies often assessed the physical activities and load of nursing work through self-reporting [6,9,12,14] or by observing activities of a few individuals and generalizing the observation to the group [19]. When physical activities and load are assessed by these methods, it may not reflect the true physical load because of the possible bias due to individual subjectivity. In this study, we assessed physical activities and load for each individual subject by using both on site observation of certain patient-handling tasks performed by NAs and self-reported data concerning perceived exertion at work.

Despite the high prevalence of LBP among NAs, which has been consistently observed in the studies of Western countries, very little information is available regarding Taiwan. With a rapidly aging population in Taiwan, there is an increasing number of nursing staff in the long-term care market. The objectives of this study were to investigate the prevalence of different measures of LBP among female Taiwanese NAs working in nursing homes and to investigate the potential risk factors associated with each of the LBP-related measure. These potential risk factors include individual characteristics, physical load, and psychosocial factors.

## Methods

### Design and study population

This study used a cross-sectional design. Because very few male NAs work in nursing homes in Taiwan, the study population comprised female NAs only. Participants were recruited from 31 nursing homes located in central Taiwan. The participating nursing homes were asked to contact NAs who were native Taiwanese, who had been employed in their current jobs for at least 1 year, who were not pregnant, and who were in full-time employment. There were 244 out of 267 eligible NAs who agreed to participate in this study (91.3% participation rate). The major reasons for non-participation were refusals and unavailability because of long leaves for vacation.

This study was approved by the Ethical Committee of Hung-Kuang University. Informed written consent was given by the participants prior to their inclusion in the study.

### Data collection

Between July and October 2005, the participants completed a self-administered questionnaire at their workplace (at a nursing station or in a conference room); a research assistant checked the returned questionnaires onsite to assure their completeness. Within two weeks after the questionnaire, each study participant was scheduled to be observed for frequency of her performance of high risk patient-handling tasks (HRPHTs), which are physically demanding, during a workday (day shift).

The questionnaire was designed to collect information on whether LBP was present during the past 12 months. Information on individual characteristics (age, height, weight, marital state, having preschool children, level of education, exercise in leisure time), work conditions (duration of employment in current work, average working hours per week), perceived physical exertion, and psychosocial load at work was also collected.

### Low back pain measures

Information on LBP was collected using a modified Nordic questionnaire [21] with a diagram of the lower back area. Those who indicated suffering from LBP (basically pain, numbness, tingling, aching, stiffness, or burning) lasting for at least one day during the past 12 months were then asked whether they ever experienced one or more of the four following LBP related measures: *pain lasting for at least one day *[3,8], *daily pain for at least 3 months (chronic LBP) *[3,12], *an intensity of pain score above 6 (intense pain) on a visual analogue scale from 0 to 9 *[10,11]; *medical care seeking (visit to a doctor or physiotherapist)*; and *sick leave because of LBP*. Thus, a total of five different measures were used to characterize LBP, although an individual case might be characterized by more than one LBP related measure.

### Physical workload assessment

Information on physical load was measured in two ways. First, a modified rating of perceived exertion was used to evaluate perceived physical exertion [22]. The rating ranged from 0 (resting) to 14 (maximal exertion). The NAs were asked, "*How physically exerting do you perceive an ordinary workday to be*?" Second, for each participant, a direct and continuous observation by an observer was conducted to count the frequency of HRPHTs during a workday (observed for at least 8 hours). The definition of HRPHTs was proposed by Menzel and associates, who grouped patient-handling tasks into three risk levels (i.e., high, higher and highest) [23]. In our study, we considered only the higher and highest risk tasks indicated by Menzel [23], and how often these higher and highest HRPHTs were performed by an NA in a normal day shift was also recorded. A total of five different manual HRPHTs were considered in this study, including *manual transfer of patients between bed/toilet and wheelchair, manual repositioning of patients in bed, manual transfer of patients between bed/wheelchair and bath cart, dressing of patients, and bathing of patients in bed (including making an occupied bed)*. Four observers were trained and standardized to perform the HRPHT counting. The frequency of the five HRPHTs performed by each NA was counted and recorded by one observer. If any one of the five HRPHT was completed by a pair of NAs, the frequency of that particular task was counted as 0.5 for each of the two NAs. (The study results of Garg et al. [24] demonstrate that even if patient-transfer tasks are completed by two NAs, the compressive force on the L5/S1 is harmful. According to this finding, if any one transfer task observed in our study was completed by a pair of NAs, we considered this task a risk to the lower back, and the frequency of that particular task was counted as 0.5 for each of the two NAs.) The sum of the frequencies of performing the five HRPHTs during a workday was then calculated as the "combined frequency of HRPHTs in a workday".

### Psychosocial workload assessment

A Chinese version of a "Job Content Questionnaire" (C-JCQ) [25], based on Karasek's demand-control-support model [26], was used to determine a study participant's psychological workload. The C-JCQ comprises 27 questions, which can be classified into four principal psychosocial aspects at work:*psychological demands, job control, social support, and job satisfaction*. Psychological demands were assessed by 5 questions. The job control scale consisted of two subscales: skill discretion (measured by 6 questions) and decision authority (measured by 3 questions). The social support scale consisted of two subscales: supervisor support and coworker support, both measured by 4 questions. Job satisfaction was assessed by 5 questions. Each question was rated on a four-point Likert scale, ranging from 1 (strongly disagree) to 4 (strongly agree). Each scale yielded a sum, with individual questions weighted according to the calculation formulas proposed by Cheng [25]. Therefore, the possible score ranges of different subscales are as follows: skill discretion, decision authority, and psychological demand scales ranged from 12 to 48; supervisor support and coworker support scales ranged from 4 to 16; and job satisfaction scales ranged from 0 to 100. The job control score was summed from the skill discretion and decision authority scores, and the social support score was summed from the supervisor and coworker support scores.

### Reliability evaluation

In a pilot study, the four observers were trained to identify and assess the five HRPHTs investigated in this study. After training and standardization, the four observers simultaneously watched a selected NA at the workplace, recording the frequency of individual HRPHT performed by this NA. The inter-observer reliability for each of the five tasks was calculated as a coefficient of variance (CV). The CVs for transfer of patients between bed/toilet and wheelchair, manual repositioning of patients in bed, manual transfer of patients between bed/wheelchair and bath cart, dressing of patients, and bathing of patients in bed were 0.95, 0.94, 0.96, 0.96, and 1, respectively. The difference among the four observers in our study was considered minimal.

To evaluate the test-retest reliability of each of the five different measures of LBP and each of the psychosocial dimensions as well as perceived physical exertion, we randomly selected 1 participant from each of the 31 nursing homes two weeks after they completed questionnaires. Of these 31 NAs, 27 NAs agreed to answer the questions again. The Kappa coefficients (κ) for the LBP lasting for at least one day, chronic LBP, intensity of pain score above 6, medical care seeking, and sick leave because of LBP were 0.83,0.71, 0.76, 0.70, and 0.76, respectively. As for psychosocial dimensions, the Pearson correlative coefficients (r) of skill discretion, decision authority, psychological demands, supervisor support, coworker support, and job satisfaction were 0.72, 0.89, 0.81, 0.78, 0.88, and 0.85, respectively. With respect to perceived physical exertion, the Pearson r was 0.82.

### Statistical analysis

For each measure of LBP, the study subjects classified as cases according to their responses to the questionnaire were compared with the rest of the sample. Odds ratios (ORs) and 95% confidence intervals (95% CIs) were calculated by separate multivariate logistic regressions with a forward selection procedure to assess the associations between potential risk factors (independent variables) and each of the five measures of LBP (dependent variables). First, variables listed in Table 1 and Table 2 were entered into the model simultaneously. Then, those variables whose coefficients were at least marginally significant (based on the likelihood ratio tests) were retained in the regression model and tested in order until the point at which all variables not in the model had a significance higher than 0.05. Smoking was not entered into these models because only 14 NAs had ever smoked. Answers to "*Having preschool children*" were omitted from the models to avoid collinearity because this item had a high correlation with marriage or cohabitation. Besides, body weight and height were omitted from the models because BMI, which is calculated by weight (kg)/height^2 ^(m^2^), was included as a risk factor. Similarly, combined frequency of HRPHTs in a working day, job control, and social support were also omitted from the models, because these items were summed from individual items, which have already been included in the regression models. The statistical analyses were conducted with the Statistical Package for Social Sciences (SPSS), version 10.

**Table 1:** Description of individual characteristics of the nursing aides (n = 244)

	n	%
Education level, lower^a^	120	49.2
Married or Cohabiting	218	89.3
Have preschool children	51	20.9
Smoking, ever	14	5.7
Exercise in leisure time^b^	129	52.9
	
	Mean	SD^c^
	
Age (years)	43.3	7.9
Duration of employment in current work (years)	6.1	4.1
Work hours in one week	47.3	4.6
Height (cm)	157.4	4.9
Weight (kg)	59.3	9.4
BMI (kg/m^2^)	24.0	3.6

^a ^Lower than high school

^b ^Exercise more than 2 hours per week, like walking, cycling, dancing

^c ^SD, standard deviation

**Table 2:** Description of work-related psychosocial and physical load for the nursing aides (n = 244)

	Mean	SD
**Psychosocial load**		
Job control	62.5	6.3
Skill discretion	32.4	3.4
Decision authority	30.1	4.8
Psychological demands	31.4	3.6
Social support	23.7	2.6
Supervisor support	11.3	1.8
Coworker support	12.4	1.5
Job satisfaction	53.3	8.7
**Physical load**		
Combined frequency of HRPHTs^a ^in a workday	58.2	13.9
Manual transfer of patients between bed/toilet and wheelchair	23.0	8.5
Manual repositioning of patients in bed	11.9	3.9
Manual transfer of patients between bed/wheelchair and bath cart	8.5	1.6
Dressing of patients	9.8	3.4
Bathing patients in bed	5.1	1.8
Perceived physical exertion score	9.1	2.1

^a ^High risk patient-handling tasks

## Results

### Characteristics of the study participants

Table 1 shows the characteristics of the 244 study participants. A majority of them were married or cohabiting (89.3%), 20.9% had preschool children, and 49.2% had a low level of education (lower than high school). The average participant was 43.3 years old, had worked 6.1 years on the current job, worked 47.3 hours per week, and had a BMI of 24 kg/m^2^. According to the above results, Taiwanese NAs working in nursing homes tended to be characterized by long working hours, a low education level, and older age but short tenure in their current job. In addition, these NAs cared for an average of 11.8 (SD = 5.4) patients during a day shift, and 89.7% of them had shift work (data not shown in table).

### Work-related psychosocial and physical load

Table 2 presents the average scores of job control, psychological demands, social support, and satisfaction, which were 62.5, 31.4, 23.7 and 53.3, respectively. Of the five HRPHTs observed in this study, the most frequent task among NAs was manual transfer of patients between bed/toilet and wheelchair, and the average frequency of combined HRPHTs in a workday was 58.2. The correlations between the frequency of the five HRPHTs performed by NAs all showed significantly positive relationships (Pearson r = 0.18–0.61). In addition, the mean score of the perceived physical exertion was 9.1, and 15.6% of these 244 NAs reported high exertion (a score > 10) according to the criteria by Josephson et al. [11].

### Twelve-month period of prevalence of LBP according to different measures

The five different LBP measures presented a wide range of prevalence rates (Table 3). The most prevalence measure (66%) was pain lasting for at least one day, which represents a broad category. Lower prevalence rates were observed for chronic LBP (8.6%) and sick leave because of LBP (10.7%), which represent relatively severe measures of LBP. It is noteworthy that all of the NAs who reported chronic LBP (21/21) also reported having sought medical care, and 76.2% also reported to suffer from intense pain. Additionally, there were 26 NAs who had been absent because of LBP; among them, 92.3% (24/26) had also perceived intense pain, and 88.5% (23/26) had also sought medical care for LBP.

**Table 3:** Prevalence of LBP during the previous 12 months, according to five different LBP related measures (n = 244)

LBP definition	n	%
LBP at least 1 day	161	66.0
Chronic LBP	21	8.6
Intense pain	93	38.1
Medical care seeking	107	43.9
Sick leave	26	10.7

### Risk factors for different measures

Table 4 shows the significant factors associated with each of the five measures of LBP. Results indicate that the different measures of LBP were found to be associated with different potential predictors, whereas at least one patient-handling task and one psychosocial factor were related with each measure of LBP. Three risk factors including manual transfer of patients between bed/wheelchair and bath cart, perceived physical exertion, and psychological demands, were often associated with different measures of LBP.

**Table 4:** Association of different LBP related measures with risk factors identified from multivariate forward stepwise logistic regressions ^a^

LBP measures	Significant risk factors	OR	95% CI	P value
LBP lasting for at least 1 day	Skill discretion score	0.81	0.72–0.91	< 0.001
	Psychological demands score	1.61	1.39–1.87	< 0.001
	Job satisfaction score	0.96	0.92–0.99	0.042
	Frequency of manual transfer of patients between bed/wheelchair and bath cart in a workday	1.75	1.36–2.26	< 0.001
	Perceived physical exertion score	1.27	1.06–1.52	0.009
Chronic LBP	Age (yr)	1.14	1.02–1.27	0.021
	Decision authority score	0.81	0.70–0.92	0.002
	Psychological demands score	1.34	1.09–1.61	0.006
	Supervisor support	0.62	0.42–0.91	0.015
	Frequency of manual transfer of patients between bed/wheelchair and bath cart in a workday	1.60	1.01–2.53	0.049
Intense pain	Psychological demands score	1.46	1.24–1.73	< 0.001
	Coworker's support score	0.90	0.81–0.99	0.041
	Frequency of dressing of patients in a workday	1.94	1.53–2.47	< 0.001
	Frequency of bathing patients in bed in a workday	2.62	1.67–4.11	< 0.001
	Perceived physical exertion score	1.99	1.46–2.73	< 0.001
Medical care seeking	Exercise in leisure time^b ^(yes vs. no)	0.41	0.18–0.93	0.032
	Decision authority score	0.88	0.79–0.97	0.011
	Psychological demands score	1.66	1.41–1.95	< 0.001
	Frequency of manual transfer of patients between bed/wheelchair and bath cart in a workday	3.51	2.33–5.28	< 0.001
Sick leave	Supervisor's support score	0.53	0.39–0.72	< 0.001
	Job satisfaction score	0.90	0.84–0.96	0.001
	Frequency of bathing patients in bed in a workday	2.00	1.17–3.41	0.011
	Perceived physical exertion score	1.48	1.08–2.04	0.015

^a ^Potential risk factors entered in each model included age, BMI, marital state, educational level, current work years, working hours per week, exercise in leisure time, skill discretion, decision authority, psychological demands, supervisor support, coworker support, job satisfaction, perceived physical exertion, frequency of manual transfer of patients between bed/toilet and wheelchair, frequency of manual repositioning of patients in bed, frequency of bathing patients in bed, frequency of dressing of patients, and frequency of bathing patients in bed.

^b ^Exercise more than 2 hours per week

None of the demographic variables and work-condition variables (working years on the current job, weekly working hours) was found to be significant risk factors for pain lasting for at least one day, intense pain, or medical care seeking. Age was the only significant demographic risk factor associated with chronic LBP, with a 13% increase in risk for an increase of 1 year of age. Additionally, more than 2 hours of exercise per week was found to be negatively associated with medical care seeking for LBP.

## Discussion

We found quite a high one-year prevalence rate (66%) of pain lasting for at least one day, as compared with findings from other larger studies using the same measure. With the same measure of LBP, a previous Taiwanese study reported a prevalence rate of 58.3% in hospital nurses [27]. Lower figures were also noted in studies conducted in Hong Kong (41.6%) [28], Italy (44%) [3], and England (45%) [8]. The prevalence rate of intense pain in our study was 38.1%, which was also higher than that noted among Swedish nursing personnel (16%) [11]. On the other hand, the prevalence rates of chronic LBP and medical care seeking observed in our study were 8.6% and 43.9%, respectively, which are similar to those found in Greek (11%, 45%) and Dutch (12%, 45%) nursing staffs [29].

For sick leave, representing a severe consequence of LBP, we noted a prevalence rate of 10.7% in our study sample, which is lower than that of nursing staffs in Greece (17%) [12] and the Netherlands (15%) [29]. The low prevalence of sick leave noted in the study sample could be related to organizational culture in Taiwan. Unlike those in Western countries, Taiwanese workers are very unwilling to take a sick leave from their work simply because being absent too often can influence their performance evaluation and even their paid wages. Also, because most nursing homes in Taiwan have a small staffs, the absence of even one NA will greatly increase the workload of the other NAs, which might further discourage NAs from taking sick leaves.

Two previous studies indicated that the risk factors for LBP differ with how the LBP is defined and measured [3,16]. In this study, we also found that different measures of LBP were associated with different sets of risk factors. Some risk factors (such as manual transfer of patients between bed/wheelchair and bath cart, perceived physical exertion, and psychosocial demands) often appeared in the final regression models acting as the independent predictors for LBP, whereas some other factors were related with only one measure. This information can be taken into consideration while any preventive strategy for LBP is considered and formulated.

This study revealed that physical factors were more strongly associated with severe LBP (including intense LBP, medical care seeking and sick leave) than with mild LBP (pain lasting for at least one day). For example, when NAs increased the number of manual transfer of patient between bed/wheelchair and bath cart by one time in a workday, the risk of pain lasting for at least one day increased by 75% (OR = 1.75), and the risk for medical care seeking for LBP increased by a much magnitude at 251% (OR = 3.51). These findings were consistent with previous findings showing that manual patient handling is the most important risk factor for LBP among nursing staff [7,8,12]. Moreover, when the perceived physical exertion in a workday increased by one point, the risk of pain lasting for at least one day increased by 27% (OR = 1.27), and the risk for intense pain and sick leave because of LBP increased by a magnitude of 99% (OR = 1.99) and 48% (OR = 1.48), respectively. Our findings also support a report that concluded that physical risk factors alone are necessary and sufficient to produce work-related musculoskeletal disorders [30]. Many past reports suggested that nursing personnel's utilization of mechanical patient lifts can efficiently reduce the risk of developing musculoskeletal disorders [31-33]. According to our on-site observations, most NAs did not use assistive devices to transfer or lift patients. They usually used traditional manual techniques such as working in pairs or even by oneself to perform such activity. One possible reason for why Taiwanese NAs seldom utilize assistive devices is that using assistive devices is more time-consuming, and NAs have only limited time to provide care to patients because the number of NAs working in nursing homes is very limited in Taiwan.

This study indicated that psychological demands were a significant risk factor for four of the five LBP related measures. Psychological demands at work forced the workers to work fast, to work hard, and to do excessive work in an insufficient amount of time [25]. An increasing number of studies have suggested that time pressure is a risk factor for musculoskeletal injuries [3,34,35]. Time pressure in work reflects insufficient staffing resources [35]. The aide-to-patient ratio in this study was 1:12 in a workday, which is a heavier workload as compared to the NAs working in Californian nursing homes with patient-to-aide ratios ranging 7.6 to 10.4. [36]. Larese and Fiorito indicated that nurses in units with high patient-to-nurse ratios had more back pain and injuries than those who worked in units with lower ratios [37]. An insufficient nursing workforce might increase the frequency of manual handling per NA per shift, and in turn lead to an increased risk of LBP.

A prospective study revealed that the most important risk factor for sick leave for NAs was perceived lack of an encouraging and supportive culture in the work unit [38]. Another prospective study also showed that low job satisfaction and insufficient supervisor support were risk factors for sick leave due to low back pain [39]. In the present study, lower job satisfaction and lower supervisor support were found to be associated with an increased risk of sick absence because of LBP. This could be due to the fact that sick leave is a way of coping with poor job satisfaction or supervisor support. In contrast, higher job satisfaction and higher supervisor support seem to change work demands, and, hence allow the NAs to more easily continue working even with LBP complaints [38,40]. In this study, NAs who suffered from intense pain or chronic pain for LBP were more likely to seek medical care. This result is no surprise, and it is similar to the findings of previous studies [29,41]. Furthermore, the study of a general working population by Mortimer and associates indicated that apart from physical and psychological factors, pain intensity and disability were the most influential factors for seeking medical care [42].

Demographic characteristics were all found to be unrelated to the risk of most LBP measures in this study. The only exception is that older age was observed to be a significant risk factor for chronic LBP. This finding is consistent with findings from a review by Burdof and Sorock [43]. The positive relation between age and chronic LBP has been established in previous studies [16,43]. However, another two studies which were both conducted in younger nursing staffs (the mean age of the two studies were 35.8 and 37 years old, respectively) presented no association between age and chronic LBP [3,12]. The mean age of NAs in this study is relatively old at 43.3 years of age, and it might suggest the existence of a degenerative process and accumulation of spinal damage [16].

To our knowledge, no previous studies have found a link between exercise and medical care seeking. Mortimer and Ahlberg noted that performing sport activities on a weekly basis was not associated with seeking medical care because of LBP [42]. Unlike the study by Mortimer and Ahlberg, we noted a significant inverse association between exercise in leisure time and medical care seeking for LBP. The NAs with a habit of regular exercise were less likely to seek medical care when their lower backs were not feeling well.

## Limitations

The cross-sectional design of this study precludes the causal influence between risk factors and various LBP related measures. In addition, a cross-sectional or self-report study might be subject to exaggeration of some relationships noted in this study since the study subjects with LBP might be inclined to over-report their psychosocial load or physical exertion [43]. Moreover, the recall period for the experience of LBP was as long as 12 months, which could also entail a certain degree of misclassification due to recall bias. The statistical relation between risk factors and chronic LBP and sick leave might be by chance due to the small number of subjects in the two categories of LBP. Finally, since this study was limited to actively employed NAs, those who left the workforce due to LBP were therefore excluded from the current analysis. Thus, the prevalence rates of LBP noted in this study might have been underestimated. The association of certain job related factors might also have been attenuated accordingly. A larger sample size and a prospective cohort study design may be warranted in the future to provide more sound research evidence.

## Conclusion

This study indicated that the prevalence rate of LBP NAs in Taiwan is high and more active steps should be taken to address this problem. Certain manual patient-transfer tasks and psychological demands seem to be among the most important risk factors for various measures of LBP in NAs. The promotion of the use of mechanical assistive devices to transfer patients, and some administrative policies to improve the psychosocial work environment, should be considered to reduce the occurrence of LBP among NAs employed in nursing homes.

## Competing interests

The author(s) declare that they have no competing interests.

## Authors' contributions

CKF participated in the design of the study, trained the observers, carried out the data collection, performed the statistical analysis and drafted the manuscript. IFM designed the study protocol and managed the coordination. MLC participated in the design of the study and revised the manuscript. All authors read and approved the final manuscript.

## Pre-publication history

The pre-publication history for this paper can be accessed here:


